# Building reliable radiomic models using image perturbation

**DOI:** 10.1038/s41598-022-14178-x

**Published:** 2022-06-16

**Authors:** Xinzhi Teng, Jiang Zhang, Alex Zwanenburg, Jiachen Sun, Yuhua Huang, Saikit Lam, Yuanpeng Zhang, Bing Li, Ta Zhou, Haonan Xiao, Chenyang Liu, Wen Li, Xinyang Han, Zongrui Ma, Tian Li, Jing Cai

**Affiliations:** 1 Department of Health Technology and Informatics, The Hong Kong Polytechnic University, 11 Yuk Choi Rd, Hung Hom, Hong Kong SAR China; 2 OncoRay – National Center for Radiation Research in Oncology, Faculty of Medicine and University Hospital Carl Gustav Carus, Technische Universität Dresden, Helmholtz-Zentrum Dresden - Rossendorf, Dresden, Germany; 3 National Center for Tumor Diseases (NCT), Partner Site Dresden, Germany: German Cancer Research Center (DKFZ), Heidelberg, Germany; 4 Faculty of Medicine and University Hospital Carl Gustav Carus, Technische Universität Dresden, Dresden, Germany; 5 Helmholtz Association / Helmholtz-Zentrum Dresden - Rossendorf (HZDR), Dresden, Germany

**Keywords:** Biomarkers, Computed tomography, Cancer imaging, Cancer models

## Abstract

Radiomic model reliability is a central premise for its clinical translation. Presently, it is assessed using test–retest or external data, which, unfortunately, is often scarce in reality. Therefore, we aimed to develop a novel image perturbation-based method (IPBM) for the first of its kind toward building a reliable radiomic model. We first developed a radiomic prognostic model for head-and-neck cancer patients on a training (70%) and evaluated on a testing (30%) cohort using C-index. Subsequently, we applied the IPBM to CT images of both cohorts (Perturbed-Train and Perturbed-Test cohort) to generate 60 additional samples for both cohorts. Model reliability was assessed using intra-class correlation coefficient (ICC) to quantify consistency of the C-index among the 60 samples in the Perturbed-Train and Perturbed-Test cohorts. Besides, we re-trained the radiomic model using reliable RFs exclusively (ICC > 0.75) to validate the IPBM. Results showed moderate model reliability in Perturbed-Train (ICC: 0.565, 95%CI 0.518–0.615) and Perturbed-Test (ICC: 0.596, 95%CI 0.527–0.670) cohorts. An enhanced reliability of the re-trained model was observed in Perturbed-Train (ICC: 0.782, 95%CI 0.759–0.815) and Perturbed-Test (ICC: 0.825, 95%CI 0.782–0.867) cohorts, indicating validity of the IPBM. To conclude, we demonstrated capability of the IPBM toward building reliable radiomic models, providing community with a novel model reliability assessment strategy prior to prospective evaluation.

## Introduction

Radiomics is a flourishing field in which machine learning is used to associate cancer imaging phenotypes with cancer genotypes or clinical outcomes for precision medicine^1–3^. Radiomics strives to characterize the differences in tumor phenotypes based on non-invasive medical images, such as computed tomography (CT), magnetic resonance imaging, and positron emission tomography. Furthermore, radiomics can be used to capture the heterogeneity of a tumor^4^, associate heterogeneity with tumor characteristics for diagnosis^5^ and treatment prognostication^6^, and improve the overall decision-making during treatment^7^.

Despite the potential of radiomics, the unknown reliability of reported radiomic features and signatures against the variability of image acquisition, reconstruction, and segmentation is one of the major challenges in translating radiomic models from bench to bedside^8,9^. Lafata et al.^10^ reported the variability of a classification model for non-small-cell lung cancer histology with respect to free-breathing 3D-CT and phases of 4D-CT imaging. In addition to radiomic model applications, the deep-learning model variability caused by variations in analyzed images should be considered. Blazis et al.^11^ reported the impact of CT reconstruction parameters on the performance of a lung nodule computer-aided diagnosis (CAD) system based on deep learning. They found that the performance of the CAD system increased when the iterative reconstruction levels or the image quality were also increased. Both publications suggest that the impact of imaging variations on the reliability of radiomic models need to be better understood.

To our knowledge, no study has compared the reliability of radiomic models with that of features—against imaging variations. Multiple scans of the same patients obtained within a short interval are necessary to conduct a model reliability study, where the predicted outcomes from different scan sets could reflect the model variability. As obtaining such datasets is resource-intensive and increases the burden on the patient, they are only obtained for research purposes. To obtain multiple datasets, Zwanenburg et al.^12^ proposed perturbing the images and contours to simulate the acquisition of multiple image sets. They validated this method by comparing the feature robustness with that in two test–retest datasets.

Following this idea, we propose a reliability assessment method of the radiomic model using perturbations. In addition to traditional radiomic modeling methods, we simulated multiple internal validation datasets by adding plausible perturbations to the original images and segmentations. The perturbed data were then used to validate the reliability of the radiomic model against randomization, and reliability was indicated by the intraclass coefficient of correlation (ICC), which was used to describe the consistency of model prediction outcomes within the same patient across all perturbations.

## Results

First, the optimal features and associated characteristics for model building are reported. Second, the model's performance on the original and perturbed dataset are evaluated. Third, the reliability of the radiomic model is computed.

**Figure 1:**
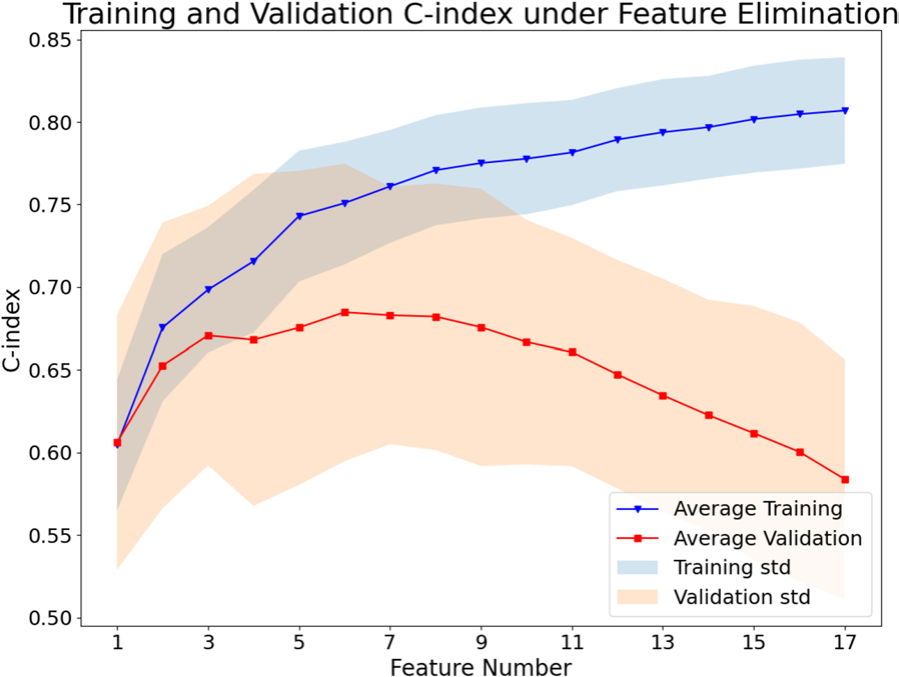
Changes in the training and validation C-indexes with respect to feature numbers in the step-wise backward feature elimination method under three-fold cross-validation, repeated 10 times. The points indicate the averaged C-index over cross-validation folds, and the shaded area indicates the range of one standard deviation (std).

The first step was to identify the features relevant to the outcome and remove redundant features. After filtering, 17 of 5486 features were selected. Then, a backward recursive feature elimination based on a penalized Cox proportional hazard model was used to find the optimal feature set for model building. Figure 1 shows the changes in training and validation C-indexes of a 10-times-repeated, three-fold cross-validation of the training dataset with respect to the number of features in the recursive feature elimination process. The feature set with the highest validation C-index was identified as the optimal feature set, and thus six features were identified as the optimal feature set and used for model building. The characteristics of these six selected features are tabulated in Table 1.

**Table 1:** The characteristics of selected features for model building.

Features	C-index	*p*-value	ICC
Log-sigma-6-0-mm-3D_gldm_LargeDependenceLowGrayLevelEmphasis_64_binCount	0.619	0.045	0.747
Wavelet-HHL_glrlm_LongRunLowGrayLevelEmphasis_128_binCount	0.587	0.169	0.454
Original_glszm_LargeAreaLowGrayLevelEmphasis_128_binCount	0.614	0.066	0.610
Wavelet-LLL_glrlm_RunEntropy_128_binCount	0.608	0.064	0.900
Wavelet-LHL_glszm_LowGrayLevelZoneEmphasis_64_binCount	0.572	0.091	0.491
Wavelet-HLL_glszm_SmallAreaHighGrayLevelEmphasis_128_binCount	0.604	0.085	0.542

The univariate C-index, *p*-value, and ICC were tabulated. Feature names indicate the feature, the bin count (if applicable), and the image used to compute it.

After identifying the six optimal features, the radiomic survival model was constructed and validated. The C-indexes of the survival radiomic model in the training and testing cohorts were 0.742 and 0.769, respectively. The averaged model performance C-indexes (standard deviation) over the perturbed training and testing cohorts were 0.686 (0.038) and 0.678 (0.065), respectively.

**Figure 2:**
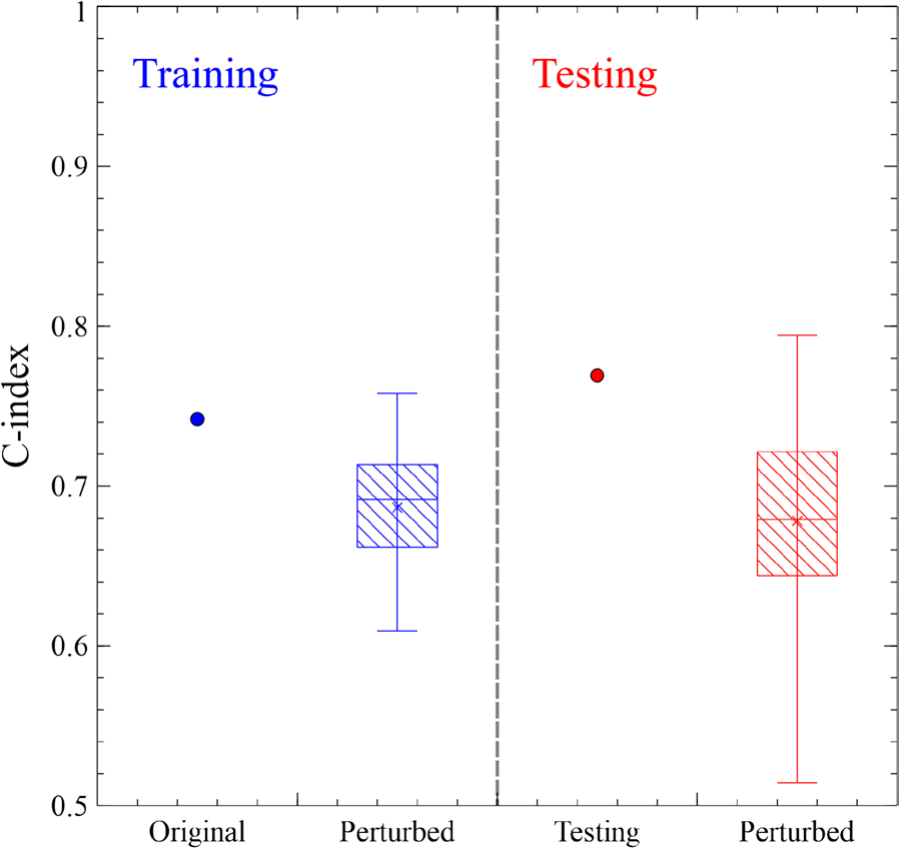
Visualization of model performance on the original and perturbed data.

The model performance on the original and perturbed cohorts is visualized in Fig. 2, which shows that the original training and testing C-indexes probably overestimate the model's performance compared with the perturbed cohort evaluation. Furthermore, the model performance variations on the perturbed cohorts are significant, with C-indexes ranging from 0.609 to 0.758 in training and from 0.514 to 0.794 in testing.

**Figure 3:**
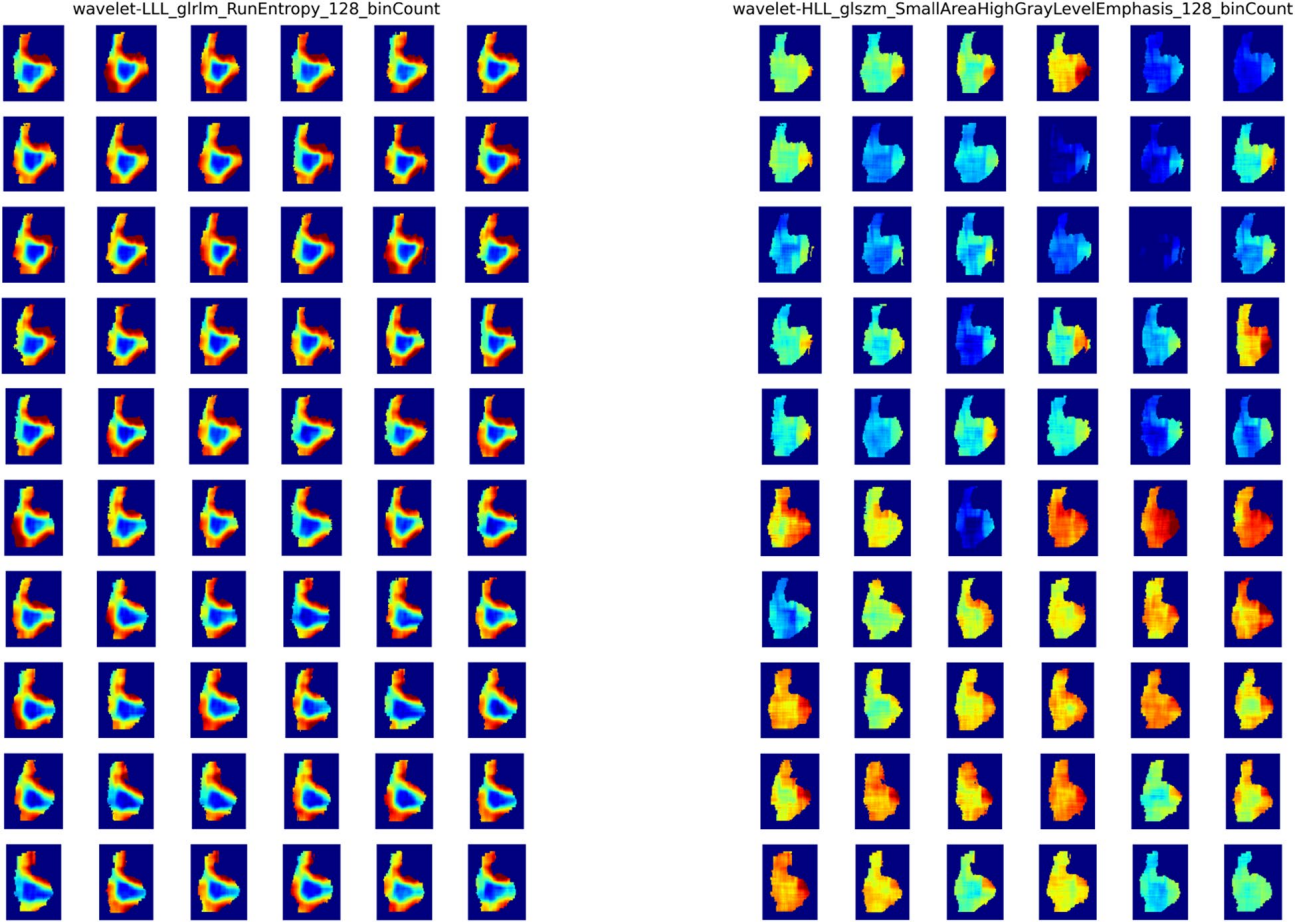
The feature map of wavelet-LLL_glrlm_RunEntropy (left) and wavelet-HLL_glszm_SmallAreaHighGrayLevelEmphasis_128_binCount (right). The window is fixed between 1 and 99 percentile of the feature map to eliminate the effects of noise.

The feature maps of feature wavelet-LLL_glrlm_RunEntropy and wavelet-HHL_glrlm_LongRunLowGrayLevelEmphasis were calculated across perturbed images to interpret the results visually. As shown in Fig. 3, the feature map of RunEntropy showed a homogeneous pattern across perturbed images than the feature map of LongRunLowGrayLevelEmphasis, which is consistent with the feature robustness ICC calculated using perturbation images.

**Figure 4:**
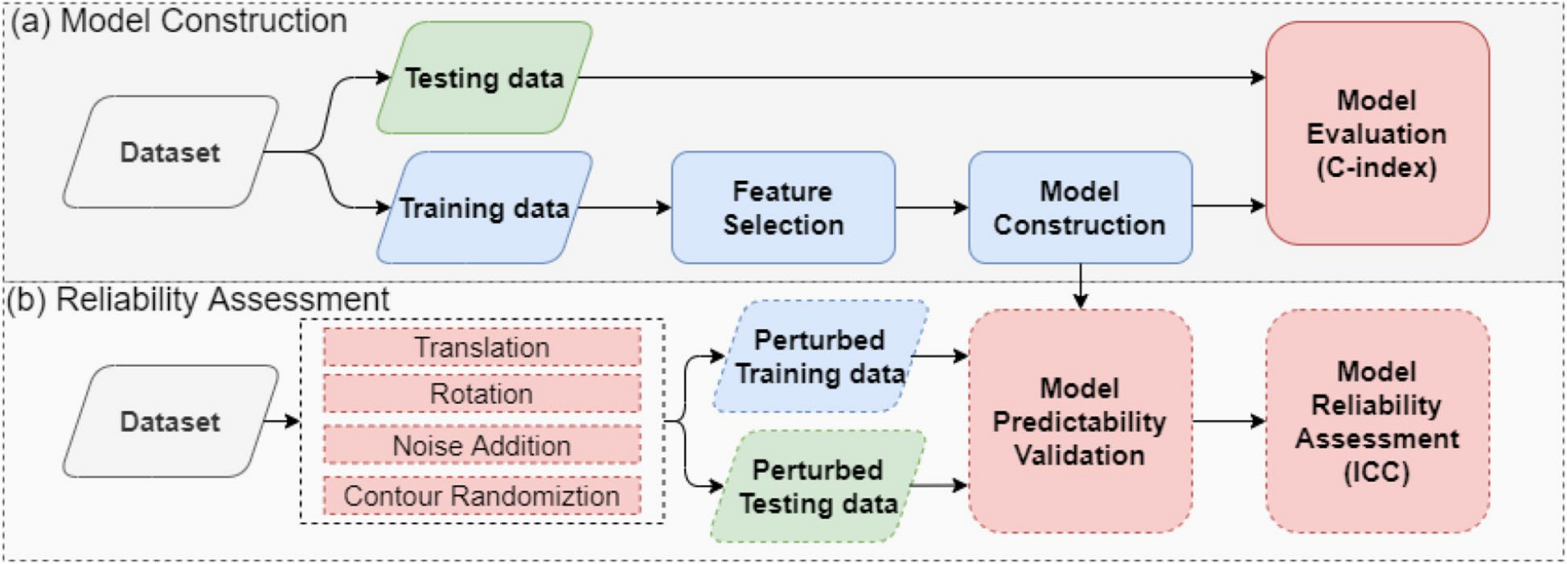
The general workflow of the study.

After evaluating the model's discriminatory power, the quantified model performance using ICC was calculated with a 95% confidence interval. The model reliability ICC was 0.565 (0.518–0.615) on the training set and 0.596 (0.527–0.670) on the testing set. According to convention^13^, this model's reliability is moderate (0.5 < ICC < 0.75), and it is consistent with the significant variations in model performance with the perturbed datasets, as shown in Fig. 3.

An additional experiment was performed to validate the sensitivity of the reliability ICC, using the highly reliable features (ICC > 0.75) to repeat the radiomic modeling process. After prescreening the reliable features, 67% (3667/5486) of features were retained; these were reduced to four optimal features for model building after feature selection. The new model performance C-indexes for the original training and testing cohorts were 0.711 and 0.641, respectively, while the averaged perturbed training and testing C-indexes (standard deviation) were 0.640 (0.029) and 0.625 (0.042). The model reliability ICC values, with a 95% confidence interval, were 0.782 (0.749–0.815) and 0.825 (0.782–0.867) for the perturbed training and testing sets, respectively. The univariable analysis result has been tabulated in Table 2.

**Table 2:** The characteristics of selected features for model building in sensitivity analysis.

Feature names	C-index	*p* value	ICC
Log-sigma-6-0-mm-3D_glszm_GrayLevelNonUniformity_128_binCount	0.653	0.00001	0.97
Wavelet-HHL_glszm_GrayLevelNonUniformity_64_binCount	0.656	0.00001	0.91

The univariate C-index, *p*-value, and ICC were tabulated. Feature names indicate the feature, the bin count (if applicable), and the image used to compute it.

An additional experiment, starting with highly reliable features, led to a significant increase in the model reliability ICC values from moderate to good. This result demonstrated the sensitivity of our method to input reliability.

The subgroup analysis based on filtered images was also performed. The median value radiomic feature ICC (range) for the original image group, log-sigma image group, and wavelet image group is 0.87 (0.42–1.00), 0.91 (0.35–0.99), and 0.77 (0.14–0.99). Table 3 showed the subgroup analysis results based on the filtered image groups. In general, the trend of improving model reliability is maintained, which also indicates that our method can be used to quantify radiomic model reliability for quantitative analysis using filtered or non-filtered images.

**Table 3:** The model performance in discrimination and reliability.

	Training C-index	Testing C-index	Model robustness ICC
**No filtering**			
Original features	0.67	0.71	0.72
Log-sigma features	0.72	0.54	0.59
Wavelet features	0.80	0.56	0.51
**Feature ICC > 0.75**			
Original features	0.65	0.74	0.85
Log-sigma features	0.58	0.54	0.91
Wavelet features	0.62	0.55	0.89

An improvement in model reliability is observed after removing non-robust radiomic features.

The cross-validation analysis was also performed to validate the generalizability of our model reliability assessment method. The averaged model reliability ICC (standard deviation) in testing cohorts over cross-validations are 0.83 (0.13), whereas the averaged training C-index (standard deviation) over cross-validation is 0.77 (0.07) and the averaged testing C-index (standard deviation) is 0.67 (0.13). The results indicates that our reported result based on single split does not have significant bias in splitting. Besides, we also performed sensitivity analysis by initially removing low-robust features (ICC < 0.75) and re-run the radiomic analysis. The averaged training and testing C-index over cross-validation are 0.75 (0.04) and 0.67 (0.08), whereas the averaged model robustness ICC over cross-validation improved to 0.93 (0.04). The improvement of the model reliability (*p* < 0.05) has shown that our method is also sensitive to the change of input reliability under cross-validation.

## Discussion

This study proposed a radiomic model reliability evaluation method using data perturbations. We demonstrated this method using a publicly available dataset and by building radiomic models to predict distant metastasis-free survival. To our knowledge, this is the first study to describe a method to assess the reliability of radiomics models based on image perturbation. Our method evaluates model reliability against randomization in a radiomic workflow using the perturbation method. This study may provide a new perspective on model assessment for the radiomic community. Our results showed that model performance can be overestimated, despite the decent model predictability achieved using an independent testing set. Moreover, simulated perturbation data can serve as an internal validation method for a model reliability assessment.

This study is also the first to assess radiomic model reliability. Currently, there is no radiomic model reliability assessment method, despite consensus on the importance of building reliable radiomic models within the community^14^. This paradox may be due to several reasons. First, the reliability of a model covers a wide range of aspects, as radiomics is a multi-step process and uncertainties may be introduced in each step^8,15^. Therefore, it is challenging to characterize the stability of radiomic models. Second, limited medical resources, such as re-scanned images, prevent the internal validation of model reliability. If multiple scanned image sets obtained over a short time interval and inter-observer delineations of different scans were available, the model could be validated internally to account for random variations in parameters such as patient positioning and inter-observer delineation. Third, it is challenging to characterize a model's reliability against controllable factors, such as different scanners and acquisition parameters, because such medical resources are inaccessible. These factors have been shown to affect radiomic feature reproducibility and, potentially, model reliability. To tackle some of these challenges, our study used the perturbation method to simulate perturbed datasets, thereby accounting for randomized factors in the radiomic workflow. For example, rotation and translation mimic variations in the patient's positioning during the scans and resampling uncertainties, noise addition mimics fluctuations in the voxel values caused by statistical uncertainties, and contour randomization mimics inter-observer uncertainty in region-of-interest delineation. These simulated datasets play a crucial role in assessing radiomic model reliability.

This study also evaluated the robustness of the model against randomness. The majority of reliability studies in radiomics publications have focused on the reproducibility and robustness of controllable factors, such as the scanner brand^16^, image acquisition parameters^17^, reconstruction kernels^18^, and preprocessing parameters^19^. However, the effects of these controllable factors can be minimized with sufficiently transparent reporting. In contrast, random and natural variations persist in every radiomic study and are difficult to address by harmonization or standardization. Therefore, understanding the impact of randomness on radiomic features and models is crucial for establishing clinical radiomic applications.

Our method also has the potential in real clinical situations. Firstly, this method could help commission the radiomic model's reliability before its routine application. The evaluation material should be the institution's past data and make sure the evaluation is institution-specific. Secondly, this method could be used to calculate the patient-specific prediction outcome variability by simulating the perturbed images of the patient. The prediction outcome variability from the perturbation features expressed the random error of the prediction outcome, which also helps the decision-making process. The key of this method is that it could help to address the random error in radiomic model development and application.

Our results revealed the vulnerability of our radiomic model to randomness. In our results, the model performance evaluation using perturbed data showed lower training and testing C-indexes for the survival model and considerable variability in its distribution under perturbations. The lower training C-index for the perturbed data reveals that evaluating models using their original data results in overfitting to noise in the original data and over-estimation of the model's learning. If a model is unable to achieve a similar performance using the same data with plausible randomization, it is unlikely that it could be translated to the clinic. Careful assessment of radiomic models' reliability is therefore essential.

A potential solution to this issue is to evaluate the reliability of features under randomization and integrate this information into radiomic modeling. Despite plenty of discussion and studies of radiomic feature robustness and reliability under various circumstances, only two methods have been implemented in a few clinical studies. The first method uses a test–retest dataset and evaluates radiomic feature reliability using two consecutive scans in a short interval, followed by incorporating this reliability into the dataset. This method may reflect realistic feature reliability under test and retest settings. However, the acquisition of test–retest imaging is rarely conducted outside of a research context, and most medical imaging datasets therefore lack complimentary test–retest image data. Although some studies have adopted the test–retest RIDER Lung dataset^20^ to assess feature robustness in an attempt to build reliable models, the generalizability of feature robustness from the RIDER Lung data to the dataset being studied has been criticized^21^. The second method assesses feature robustness using inter-observer variability on the contours. The region of interest on the images is delineated multiple times by independent oncologists, and feature robustness is evaluated from the inter-observer consistency of feature values. This method is more practically accessible than test–retest images to assess feature robustness. However, this method also has limitations in terms of the insufficient identification of non-robust features and high medical personnel costs. The shortcomings of these two methods for assessing feature robustness limit their effectiveness for removing non-robust radiomic features during radiomic modeling, potentially resulting in radiomic models that are vulnerable to randomization. Therefore, simulated randomization of a dataset via the perturbation method may enable estimation of the impact of randomness on radiomic modeling. Multiple perturbed datasets can be generated with perturbations, and their feature values can be determined. Feature robustness can be quantified using the ICC for each feature by considering its variability within a single subject and across the dataset. Then, removing the less reliable features can improve the reliability of radiomic models against randomizations. In contrast to test–retest and inter-observer variability, simulation methods may be more versatile for evaluating feature robustness with no additional clinical resource costs and could enable data-specific feature robustness evaluations. Moreover, perturbations can provide additional validation data to evaluate model reliability and safeguard it against randomization.

In addition to these contributions, some aspects of our approach could be explored to enhance the impact of this study. First, image and contour perturbation via simulation is a new method in radiomics, so comparisons between this and established methods (e.g., test–retest and inter-observer variability) could be studied further to identify their respective advantages and disadvantages. Second, our validation results showed a decline in model predictability performance from the testing data when poorly and moderately reliable features were removed. A future study could investigate how to balance the model's predictive performance with its reliability. Furthermore, it is also worth mentioning the sensitivity of the model robustness after feature robustness screening is relative to the characteristics of the original feature group. For example, the median (range) feature robustness ICC in the morphological category is 0.99 (0.94–1.00) among 14 features. The median (range) feature robustness ICC in the intensity category is 0.89 (0.14–0.99) among 216 features. The median (range) feature robustness ICC in the texture category is 0.82 (0.16–1.00) among 5256 features. The improvement of model robustness after filtering is likely to be more significant in the intensity and texture features categories. In addition, this study lacks a comparison between the perturbation method and the test–retest method. Although the perturbation method has been proved to replace test–retest data in the feature level, further studies comparing the perturbation method and test–retest method in the radiomic model level is warranted in the future.

## Conclusions

This study proposed a radiomic model reliability assessment method using perturbations. This method identifies unreliable models by comparing the model's performance on the training dataset with the performance achieved on random perturbations of the training dataset. Using this approach could help the radiomics community to build more reliable models for future clinical applications.

## Materials and methods

### Overview

The overview of the workflow used to demonstrate our model reliability assessment method is illustrated in Fig. 4. First, we collected pre-treatment CT images and clinical outcomes from a publicly available head-and-neck cancer (HNC) dataset and randomly split the data into training (70%) and testing cohorts (30%), with similar outcome ratios between the two cohorts. Second, a radiomic survival model was built to assess distant metastasis-free survival. Third, internal validation datasets (Perturbed-Train and Perturbed-Test) with perturbations were simulated^12^. The simulated perturbation datasets were used to extract perturbed radiomic features and validate the survival model's reliability against randomizations, as shown in Fig. 4b. Finally, the ICC was used to quantify the model's reliability, reflecting its prediction consistency when using the perturbed data. The experiment is approved by the department of health technology and informatics, the Hong Kong Polytechnic University. The developing and reporting method of the radiomic survival model are carried out in concordance with transparent reporting of a multivariable prediction model for individual prognosis or diagnosis (TRIPOD)^22^. All methods were carried out in accordance with relevant guidelines and regulations.

### Materials

The dataset, Head-Neck-PET-CT^14^, was collected in The Cancer Image Archive^23^. This dataset consists of 298 patients with head-and-neck squamous cell carcinoma (HNSCC) with a median follow-up of 43 months. The patients were treated at four different centers and received only radiation (n = 48, 16%) or chemo-radiation (n = 250, 84%) with curative intent. The patients' characteristics and image reconstruction parameters are summarized in Supplementary Tables 1 and 2. Due to the nature of the retrospective study and the publicity of the dataset, the informed consent was waved by the Institutional Review Board of the Hong Kong Polytechnic University.

The region of interest for feature extraction was the primary gross tumor volume (GTV), which was the primary treatment target of radiation therapy. The GTV is the most reliable region for predictive feature extraction^24^ and has been used in several predictive radiomics studies of HNSCC^1,25,26^.

Distant metastasis-free survival, defined as the interval from the first day of treatment to the date of the event, was the clinical endpoint in this study to demonstrate the reliability assessment of the radiomic model^27^. Previous studies of binary classification models of HNC^25,28^ have achieved good prediction results but were limited because the time-to-event was neglected during model development.

### Image preprocessing and radiomic feature extraction

The CT images and their GTV contours were preprocessed before their features were extracted to maintain the features' reproducibility and consistency^29,30^. First, the GTV contours were interpolated to a voxel-based segmentation mask. Second, an isotropic resampler (1 mm × 1 mm × 1 mm) was applied to the images and masks, with B-spline interpolation on the image and nearest-neighbor interpolation on the mask to enhance the reproducibility of the radiomic features^31^. The preprocessing steps were implemented on Python v3.8 using the SimpleITK v1.2.4^32^ and OpenCV^33^ packages.

The radiomic features were then extracted using the Pyradiomics v2.2.0^34^ package, which is Image Biomarker Standardization Initiative-compliant^35,36^. A total of 5,486 radiomic features were extracted from the GTV of each patient's CT scan. Twelve images were included in the feature extraction, including one unfiltered image, three Laplacian-of-Gaussian filtered images (with sigma values of 1 mm, 3 mm, and 6 mm), and eight Coiflet1 wavelet filtered images (LLL, HLL, LHL, LLH, LHH, HLH, HHL, HHH). In addition to the 14 shape features from GTV segmentation, 18 first-order and 73 s-order features were extracted from the region of interest of each filtered image. A re-segmentation of the soft-tissue range (− 150 to 180)^12^ and discretization, with fixed bin counts of 4, 8, 16, 32, 64, and 128, were specified for the texture feature extraction. The detailed feature extraction parameters can be found in Supplementary 3.

### Radiomic modeling

Patients were randomly assigned to the training and testing cohorts (70/30 split) with stratification by distant metastasis status^6,37^. The data in the training cohort were used for feature selection and subsequent model training, while the data in the testing cohort were used to evaluate the model's performance. The radiomic features are standardized using the z-score method using a training cohort before feature selection to assure a similar scale in training and testing data.

### Feature selection

A filter-based feature selection method was adopted in our analysis^38^. This process has two steps: feature–outcome relevance filtering and feature–feature redundancy filtering. Identifying the most relevant and less redundant features is a common practice in radiomics studies, regardless of the evaluation metric^39^.

#### Relevance filtering

Relevance filtering aims to identify the radiomic features that are correlated with the outcomes^25^. First, the outcome relevance of each feature was repeatedly evaluated by log-rank test *p*-values under downsample bootstrapping (imbalanced-learn 0.8.0^40^) without replacement over 100 iterations on the training dataset. Downsampling can be used to capture useful information in an imbalanced dataset^41^. Second, features with *p*-values less than 0.1 were selected in each iteration and ranked by their frequencies, with the top 10% of features with the highest frequencies selected.

#### Redundancy filtering

Redundancy filtering aims to remove features correlated with each other^42^. First, the feature pairs with Pearson correlation coefficients higher than 0.6 were identified. Then, the features with higher mean correlation coefficients than the rest of the features were removed. The removal of these redundant features should improve the predictive ability of the classifiers^43^.

### Model building

To build the survival model, the optimal features for the model building were identified using backward recursive feature elimination based on the penalized Cox proportional hazard model^44^. This approach maximizes the validation concordance index (C-index) curve by using repeated three-fold cross-validation in the training set. After identifying the optimal features, a penalized Cox proportional hazard survival model was built for distant metastasis-free survival. The hyperparameter of the model was fine-tuned with five-fold cross-validation to maximize the C-index for the survival model. Thus, the model's performance with the training and testing cohorts was evaluated.

### Reliability assessment

This section describes the method to evaluate the model reliability using perturbations and the workflow shown in Fig. 4b. First, the internal validation datasets were simulated with the perturbations by adding plausible randomizations to the original images and segmentations. Second, the survival model was evaluated using both the perturbed training and testing data. Third, the model reliability against simulated randomization was quantified using the reliability index ICC.

#### Validation data simulation

The internal validation data sets were simulated using the perturbation method^12,45^. For each perturbation, both the image and mask were translated and rotated simultaneously by a random amount. This simulation aimed to mimic variations in the patient's position during imaging. Then, a random Gaussian noise field was added to the image to mimic the noise level variations between different image acquisitions^46^. All the perturbed datasets are simulated before preprocessing, and the perturbation model for each simulation is purely random. The detailed perturbation parameters are presented in Supplementary Table 3. Next, the GTV mask was also perturbed by a randomly generated deformable vector field, which aimed to simulate uncertainties in inter-observer delineations on the same target^47^. The parameters of generating deformable vector fields were tuned to ensure the simulation of plausible randomized GTV contours. The averaged dice similarity coefficient and the averaged Hausdorff distance over perturbed patients are around 0.85 and 5 mm respectively. In total, 60 sets of perturbed images and contours were simulated, with the corresponding radiomic features extracted as the internal validation sets to evaluate the model reliability under randomization.

#### Model validation

The model performance was validated and reported on the original and perturbed datasets using the C-index as the evaluation metric. Two observations may warrant attention. First, the model performance consistency between the original and perturbed datasets might be a qualitative indicator of model performance reliability against the simulated randomizations. Second, the model performance variance with perturbed datasets may reflect the model's sensitivity to slight fluctuations. A qualitative assessment of model reliability could be performed by comparing the model performance on the original and perturbed data.

#### Model reliability quantification

In addition to the qualitative analysis of model reliability, a quantification metric, the ICC, was proposed to evaluate model reliability under randomization. The ICC is often used as a reliability index for inter-rater reliability analysis^48^, and several radiomic studies have used this measure to quantify feature reproducibility^13,49,50^.

The model reliability ICC reflects the extent to which the measurements can be replicated. We aimed to determine whether model predictions can be repeatedly measured/produced after adding plausible randomizations to the images and segmentations both for the same patient and across the entire dataset. As each perturbed dataset was simulated randomly and the model was expected to yield an identical outcome, the one-way random effects with absolute agreement, ICC(1, 1), were calculated to quantify the model's reliability, with patients as the subjects and perturbations as the raters^48^. ICC values range between 0 and 1, with values closer to 1 representing more robust reliability. Typically, ICC values less than 0.5, between 0.5 and 0.75, between 0.75 and 0.9, and greater than 0.9 indicate poor, moderate, good, and excellent reliability, respectively^48^.

#### Model reliability validation

To validate the calculation of model robustness, the same experiment was repeated with highly reliable features (ICC > 0.75). This validation aimed to verify the sensitivity of the ICC in response to changes in model input reliability. An increase in feature robustness was expected to increase the model ICC. It is likely that observation in improved model robustness could be expected due to the removal of low robust features.

#### Subgroup analysis for filtered images

A subgroup analysis for each filtered image family is performed to validate if our method can be implemented in the radiomic analysis with filtered image features. Furthermore, many radiomic studies only used features from original images, and this subgroup analysis can also validate if the method is appropriate in current radiomic studies.

#### Cross-validation analysis

A cross-validation analysis is performed to validate the generalizability of our method under different train-test splits. A stratified threefold, 20 repetitions cross-validation was used to split patients into training and testing pairs. And the same modeling and evaluation method were used. The model's discriminatory power was evaluated using averaged C-index and the model's reliability was quantified using averaged ICC.

## Supplementary Information

Supplementary Information.

## Data Availability

The raw image dat is available in the cancer image archive (https://wiki.cancerimagingarchive.net/display/Public/Head-Neck-PET-CT). Our analysis data is shared in Github repository (https://github.com/vivixinzhi/Building-Robust-Radiomic-Model-Using-Perturbation).
