# Supplementary material for: Building reliable radiomic models using image perturbation
Source: Sci Rep. 2022 Jun 16;12:10035. doi: 10.1038/s41598-022-14178-x (PMC9203573; doi:10.1038/s41598-022-14178-x)
Supplement: Supplementary file 1 — Supplementary Information. [file 41598_2022_14178_MOESM1_ESM.docx]

**Identifying Reliable Radiomic Models using Image Perturbation**

**Running Title:** Radiomic Model Reliability Assessment

Supplementary Materials

1. **Patients Characteristics**

Table 1 Patients characteristics of the HN_PETCT dataset, adapted from (Vallières et al., 2017).

| Characteristics | | Number |
| --- | --- | --- |
| Total |  |  |
|  | Number | 298 |
| Gender |  |  |
|  | Male | 227 |
|  | Female | 71 |
| Age |  |  |
|  | Range |  |
|  | Mean (STD) |  |
| Tumor type | |  |
|  | Oropharynx | 205 |
|  | Hypopharynx | 12 |
|  | Nasopharynx | 28 |
|  | Larynx | 44 |
|  | Unknown | 9 |
| T-Stage |  |  |
|  | T1 | 39 |
|  | T2 | 110 |
|  | T3 | 93 |
|  | T4 | 46 |
|  | Tx | 10 |
| N-Stage |  |  |
|  | N0 | 59 |
|  | N1 | 40 |
|  | N2 | 180 |
|  | N3 | 19 |
| TNM-Stage | |  |
|  | Stage I | 4 |
|  | Stage II | 27 |
|  | Stage III | 61 |
|  | Stage IV | 204 |
|  | N/A | 2 |
| HPV status | |  |
|  | Positive | 78 |
|  | Negative | 41 |
|  | N/A | 179 |
| Treatment | |  |
|  | Radiation only | 49 |
|  | Chemo-radiation | 249 |

1. **Imaging and Reconstruction Details**

Table 2 Parameters for the CT characteristics in imaging and reconstruction.

| Number of patients | Exposure (mAs) | Energy (kVp) | Inplane resolution (mm^2^) | Slice thickness (mm) |
| --- | --- | --- | --- | --- |
| 92 | 12 | 140 | 0.98 x 0.98 | 3.75 |
| 102 | 210 | 140 | 1.17 x 1.17 | 3 |
| 41 | 11 | 140 | 3.52 x 3.52 | 3.75 |
| 65 | 350 | 140 | 0.98 x 0.98 | 1.5 |

1. **Feature Extraction Details**

The image features were extracted with Pyradiomics, which is compliant with Image Biomarker Standardization Initiative. A total of 5486 features were extracted, the feature extraction parameter file was found in Github Respiratory after publications.

The detailed parameters are:

- Shape: the intensity volume histogram was constructed as for images with discrete, defined (non-arbitrary) image values
- Grey level co-occurrence matrix: the matrix was calculated with 𝛿 = 1 in 3D for 13 angles (26 conectivitivity). The matrix was also not distance-weighted and symmetric.
- Grey level run length matrix: the matrix is calculated in 3D for 13 directions. The matrix was also not distance-weighted.
- Grey level size zone matrix: the matrix was calculated for the entire 3D volume in a single grey level distance zone.
- Neighbourhood grey tone difference matrix: the matrix was calculated for the 3D volume with a single neighbnourhood grey tone level with 𝛿 = 1.
- Grey level distance zone matrix: the matrix was calculated in a single grey level distance zone for the entire 3D volume.

The parameters for feature calculations are the default settings of pyradiomics packages.

1. **Image Perturbation Parameters**

Table 3 The perturbation parameters.

| Perturbation modes | Perturbation range | Reference axis | Perturbation number | Total number |
| --- | --- | --- | --- | --- |
| Translation distance (mm) | 0 to 3 with a 0.2 step size | AP, SI, LM | 4,096 | 4,423,680 |
| Rotation angles (degree) | -20 to 20 with a 5 step size | SI | 9 |  |
| Noise addition level | 0, 1, 2, 3 | - | 4 |  |
| Contour Randomization | 30 | - | 30 |  |

1. **Data Availability**

The raw image dat is available in the cancer image archive (<https://wiki.cancerimagingarchive.net/display/Public/Head-Neck-PET-CT>). Our analysis data can be found in Github (<https://github.com/vivixinzhi/Building-Robust-Radiomic-Model-Using-Perturbation>).
